# Targeted Clindamycin Delivery Systems: Promising Options for Preventing and Treating Bacterial Infections Using Biomaterials

**DOI:** 10.3390/ijms25084386

**Published:** 2024-04-16

**Authors:** Dagmara Słota, Josef Jampilek, Agnieszka Sobczak-Kupiec

**Affiliations:** 1 Department of Materials Science, Faculty of Materials Engineering and Physics, KrakowUniversity of Technology, 37 Jana Pawła II Av., 31-864 Krakow, Poland; agnieszka.sobczak-kupiec@pk.edu.pl; 2 Department of Analytical Chemistry, Faculty of Natural Sciences, Comenius University, Ilkovicova 6, 842 15 Bratislava, Slovakia; 3 Department of Chemical Biology, Faculty of Science, Palacky University, Slechtitelu 27, 783 71 Olomouc, Czech Republic

**Keywords:** clindamycin, drug delivery systems, antibiotic, surgical site infection, biomaterials

## Abstract

Targeted therapy represents a real opportunity to improve the health and lives of patients. Developments in this field are confirmed by the fact that the global market for drug carriers was worth nearly $40 million in 2022. For this reason, materials engineering and the development of new drug carrier compositions for targeted therapy has become a key area of research in pharmaceutical drug delivery in recent years. Ceramics, polymers, and metals, as well as composites, are of great interest, as when they are appropriately processed or combined with each other, it is possible to obtain biomaterials for hard tissues, soft tissues, and skin applications. After appropriate modification, these materials can release the drug directly at the site requiring a therapeutic effect. This brief literature review characterizes routes of drug delivery into the body and discusses biomaterials from different groups, options for their modification with clindamycin, an antibiotic used for infections caused by aerobic and anaerobic Gram-positive bacteria, and different methods for the final processing of carriers. Examples of coating materials for skin wound healing, acne therapy, and bone tissue fillers are given. Furthermore, the reasons why the use of antibiotic therapy is crucial for a smooth and successful recovery and the risks of bacterial infections are explained. It was demonstrated that there is no single proven delivery scheme, and that the drug can be successfully released from different carriers depending on the destination.

## 1. Introduction

Controlling the kinetics of drug release by using drug delivery systems (DDSs) brings a number of benefits to patient health. First and foremost, they allow the delivery of the active ingredient to the exact site affected by the disease. Moreover, DDSs improve the effectiveness of the drugs, reduce the severity of side effects, and allow the use of a lower dose of the substance [1,2]. Given the above, this is one of the hottest topics in modern pharmacology or materials engineering. According to a Fortune Business Insights report, the global drug delivery systems market size was valued at $39.55 billion in 2022. It is assumed that at a sustained compound annual growth rate (CAGR) of 9.1%, the value will grow to $78.76 billion in 2030. North America holds the largest share of the market, more than 40% [3]. The DDSs market continues to grow. The main drivers are an increase in patient awareness of advanced systems that allow the selective delivery of a specific drug to a certain site with improved accuracy. This enables faster onset of the effect and reduces the risk of side effects. Also, advances in medicine and science have increased the availability of new systems and improved patient comfort.

This brief review discusses various routes of drug administration into the body, pointing out their advantages and disadvantages. The focus was placed on ceramic, polymeric, and composite clindamycin delivery systems presented in the literature for biomedical applications such as bone tissue replacement, wound healing, and acne treatment. In order to conduct the presented literature review, a search of the Google Scholar and Science Direct databases was performed. Only publications discussing the methods of manufacturing of clindamycin carriers were considered. The results of papers published between 2008 and 2024 were found, with the largest number of literature reports on the subject being between 2020 and 2022. The databases were searched based on keywords such as clindamycin; ceramic; polymer; composite; drug carrier; DDS.

The aim of this review is to identify the potential for the development and application of a specific drug in targeted therapies. Clindamycin is a broad-spectrum antibiotic; however, until now, no other literature reviews on this topic have been found, as most of the available reviews focus on a general group of drugs/active ingredients rather than on a single specific one. This brief literature review will introduce the reader to the world of clindamycin carriers, indicating the possibility of producing new multifunctional biomaterials for both hard and soft tissues.

## 2. Routes of Drug Administration and Drug Delivery

**Figure 1:**
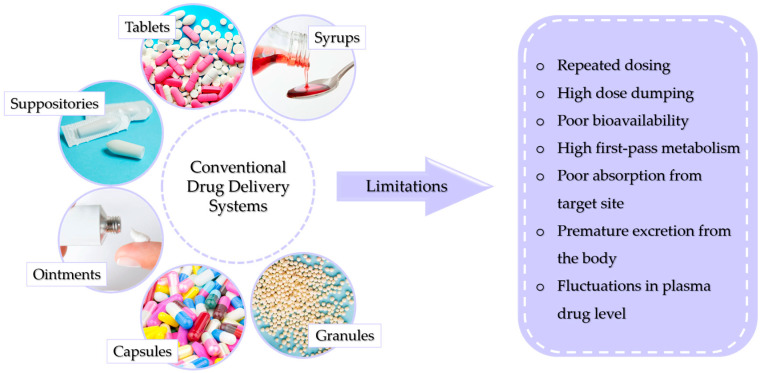
Possible limitations of conventional drug delivery systems.

There are many ways to conventionally administer drugs and active substances into the body. This can be performed using syrups, tablets, suppositories, or ointments. The dosage form is chosen depending on the physicochemical properties of the drug as well as the body part being treated, the mechanism of action of the drug, and the solubility and permeability of the substance. Conventional methods of drug administration do not allow patients to fully benefit from the therapeutic potential of the drug. This is caused by the overall distribution of the drug throughout the body. This reduces the probability of a large amount of the dose reaching its destination, requiring a higher dose to be used, which increases the risk of toxicity. Figure 1 presents conventional drug delivery methods and their main disadvantages [4].

The most popular is the oral route through which capsules, tablets, syrups, or granules are administered. This way is relatively inexpensive, the substance can be administered easily, without pain, and the absorption of the substance can occur through the entire length of the digestive tract [5,6]. However, this way is also fraught with the risk of drug destruction by stomach acid or digestive juices. The drug can also irritate the gastric mucosa and cause nausea or vomiting [7,8]. The first-pass effect, by which orally absorbed substances are initially transported to the liver via the portal vein, is also important, as it reduces drug activity [9]. In the case of ointments, the main limitation of their application is insufficient skin permeability of drugs, which reduces the therapeutic effect. Another limitation associated with ointments is that prolonged perfusion can cause membrane degradation, which can cause skin irritation [10]. In contrast, DDSs exhibit a number of advantages over conventional intake, allowing a lower dosage, providing a longer duration of action, maintaining more uniform plasma levels, or reducing the frequency of dosing [11].

Controlled drug delivery systems for targeted therapy offer the use of multiple carriers depending on the nature of the substance being delivered or the site of action. This type of therapy, also called molecularly targeted therapy, is one of the main cancer treatments since it can be applied directly to tumor cells. However, the term is also used in the case of the targeted delivery of other drugs [12]. Depending on the need and location, this process can be based on the mechanism of active and passive targeting. The first focuses on the precise targeting of diseased cells (e.g., cancerous cells) by appropriate ligands, while in contrast, in passive targeting, the drug or other substance is delivered within the perimeter of the site requiring a therapeutic effect [13]. The release of the substance from the carrier itself can occur as a result of external impulses like an electrical signal, but also as a result of a change in the pH value or swelling [14,15].

**Figure 2:**
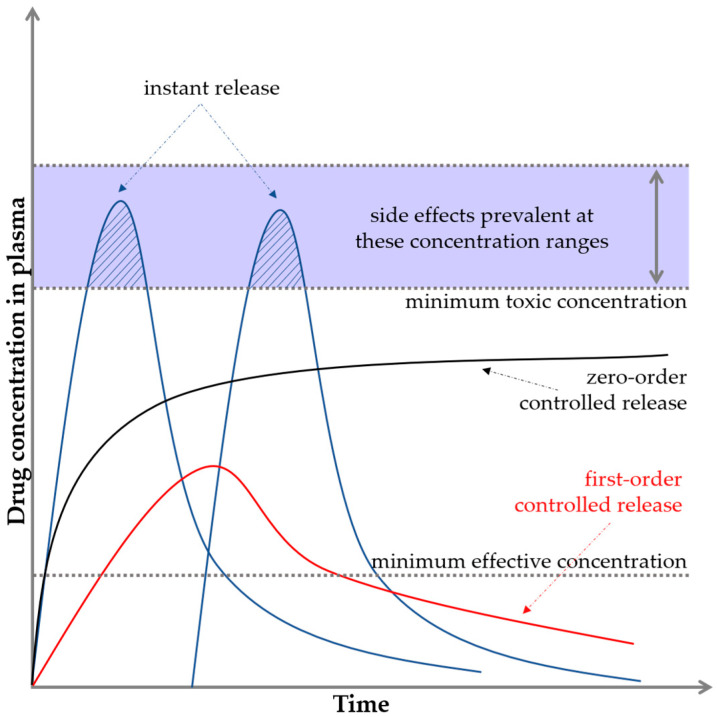
Basic pharmacological/biopharmaceutical profiles and levels.

Polymers, ceramics, and composites can be used as DDSs; however, they require appropriate processing and combination with the active substance. They include micelles, nanocapsules, microcapsules, liposomes, dendrimers, and hydrogels. The drug can be absorbed on the carrier, encapsulated in it, or bound by a chemical bond. Accordingly, the release can occur at different rates [16,17]. The drug release profile is usually expressed as the plasma drug concentration as a function of time. Figure 2 illustrates two important limits indicating the minimum and maximum concentrations.

If the drug concentration is too low, no therapeutic effect is observed, and this level is referred to as the minimum effective concentration. If the drug concentration is too high, toxicity problems may occur, and this level is known as the minimum toxic concentration [18,19]. In the case of conventional drug delivery, e.g., in tablet form, a sinusoidal shape of the graph is observed, where for a while, the limit of the maximum concentration is exceeded. This fact often causes the appearance of side effects. Then, the concentration drops sharply down below the minimum limit. The ideal situation is to keep the release of active substances in the interval between the minimum effective and minimum toxic concentrations, that is, in the so-called therapeutic window (black curve on the graph). This is crucial for maintaining the safety but also the effectiveness of the substance or drug. This is an example of zero-order drug release kinetics, which means that a constant amount of the active ingredient/drug is released per unit time; however, the rate itself does not depend on the concentration. This means that the drug is released at a constant rate [4,20,21,22].

## 3. Delivery of Antibiotics

The delivery of antibiotics in targeted therapy is critical to the risk of bacterial infections including surgical site infections (SSIs). In fact, SSIs are one of the most common infections that can occur both during hospitalization and after hospital discharge [23]. The etiologic agent leading to the infections is most often bacteria residing on the skin, but they can also be microorganisms residing in other areas of the body or found in the operating room environment as well as on surgical instruments [24,25,26]. Furthermore, bacterial infections can lead to soft tissue inflammation or osteomyelitis, which is defined as an inflammatory process caused by a bone infection that leads to bone destruction, bone necrosis, and can progress to a chronic condition [27,28]. In the case of the skin, bacteria can cause irritation, aggravate acne, or inhibit the healing process of open wounds [29]. According to the procedure recommended by the World Health Organization, antibiotic therapy can effectively prevent infections and thus problems in soft and hard tissue regeneration. For this reason, biomaterials are being developed to deliver the drug directly to the site requiring a therapeutic effect. Depending on the type of bacterial strain, different antibiotics may be used [30,31].

### Clindamycin

**Figure 3:**
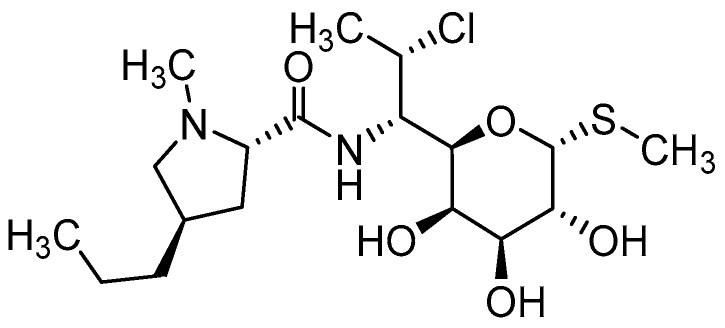
Chemical structure of clindamycin.

Clindamycin (CLD) (Figure 3) is an antibiotic from the lincosamide group used against many types of bacteria thanks to its unique properties and broad effect [32,33]. It is usually available either as a readily water-soluble salt (clindamycin hydrochloride) or as a lipophilic ester (clindamycin phosphate). This ester is a prodrug of clindamycin, which is rapidly hydrolyzed by esterases to active clindamycin after application [34,35].

The mechanism of action of CLD is based on blocking the 23S ribosomal RNA of the 50S subunit by the inhibition of peptide bond formation, which leads to the inhibition of bacterial protein synthesis [36]. For this reason, CLD is effective in treating infections caused by Gram-positive bacteria, such as *Staphylococcus aureus* and *Streptococcus pneumoniae* [37,38]. The first of them is one of the most common causes of morbidity and mortality from an infectious agent worldwide and is often associated with SSIs [39,40]. The second one is particularly dangerous for children and can cause otitis media, pneumonia, rhinosinusitis, bacteremia, and even meningitis [41].

One of the distinctive features of CLD is its ability to penetrate tissues and body fluids [42]. This makes it highly effective in the treatment of bone disorders. It not only combats infections at the superficial level, but also penetrates into areas where other antibiotics may be less effective [43,44]. For this reason, it is often used during bone transplants or other surgeries involving this tissue [45]. Additionally, CLD easily crosses the human placenta, but does not cross the blood–brain barrier [46,47]. However, it can cause side effects such as loss of appetite, abdominal discomfort, diarrhea, and nausea [32]. It is worth mentioning that clindamycin has relatively good stability under acidic conditions, which allows it to function effectively in the acidic environment of the stomach. This is important, especially when treating gastrointestinal infections, where substances can be exposed to stomach acid [48,49]. This means that in the case of this drug, conventional ingestion of CLD in tablet form is possible, as the substance will not be degraded/decomposed. Another issue is the above-mentioned limitations related to systemic distribution, which may reduce its effect [50,51]. The most significant benefits and negative effects associated with CLD administration are summarized in Table 1 [32,36,52,53].

## 4. Targeted Clindamycin Delivery Systems

### 4.1. Ceramic Carriers of Clindamycin

In order to achieve an appropriate biological effect, it is extremely important to choose the right materials for biomedical applications. In the aspect of biomaterials for bone tissue, the most commonly used are calcium phosphates, belonging to the family of biocompatible apatites. Several ceramics can be distinguished among them, which differ in their Ca/P molar ratio and thus in their properties [54,55,56]. The most popular is hydroxyapatite (HAp, Ca_10_(PO_4_)_6_(OH)_2_), which is the ceramic material most similar to the mineral phase of natural bone tissue. It has a Ca/P ratio of 1.67 and is bioactive and osteoconductive [57,58]. Another structure of calcium phosphate is calcium triphosphate (TCP, Ca_3_(PO_4_)_2_), which has osteoinductive properties and the ability to resorb under body conditions. Its molar ratio of Ca/P is 1.5. TCP and HAp are the most commonly used ceramic materials in biomaterials with the potential to regenerate or fill bone tissue [59,60]. However, brushite (DCPD—dicalcium phosphate dihydrate, CaHPO_4_·2H_2_O) or monethite (DCPA—dicalcium phosphate anhydrous, CaHPO_4_), which have the same Ca/P molar ratio of 1.0, are also used for this purpose, but differ in the amount of bound water [61,62]. Nevertheless, they are all able to provide suitable conditions for the ingrowth of bone-forming elements.

**Figure 4:**
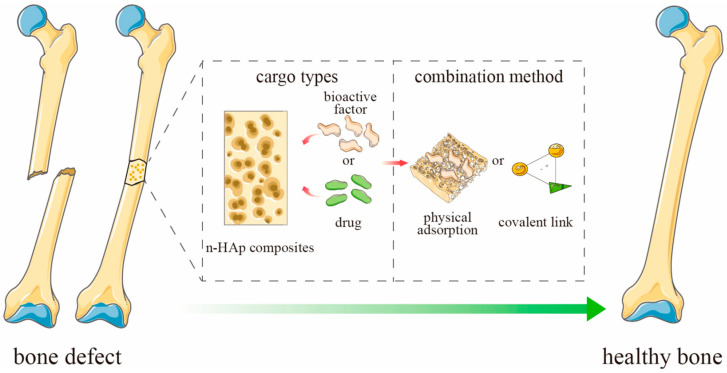
Schematic representation of ceramics-based scaffold loaded with active substances for bone defect. Adapted from [65], MDPI, 2023 (n-HAp = nano-hydroxyapatite).

The amount of drug adsorbed in the ceramic material depends on a number of factors, e.g., the concentration of the drug and the size of the carrier. However, one of the most important factors is porosity, which determines the specific surface area and affects the diffusion of the drug [63,64]. The active substance can be bound to the drug by physical sorption or by forming a covalent bond (Figure 4). The release process for most substances and porous ceramic carriers will be similar. As a result of the penetration of the liquid medium deep into the pores of the material, a systematic release of the drug into the medium occurs [56,65,66,67].

The effect of the drug release rate was compared depending on the type of ceramic. HAp and amorphous calcium phosphate (ACP) were used for this purpose. The authors compared material modified with a range of antibiotics including ampicillin, kanamycin, oxacillin, vancomycin, and clindamycin. ACP and HAp demonstrated similar levels of activity against Gram-negative organisms; however, ACP proved to be more effective against Gram-positive organisms. This suggests that the degree of crystallinity may be one of the key factors influencing antibiotic activity [68]. An example of Gram-positive bacteria against which other carriers have been tested is *S. aureus*, which infected an osteoblastic line. HAp and DCPA were subjected to drug modification through a process of physical sorption and particle aggregation induced by drying, which led to the formation of microscopic blocks. In this study, not only was the antimicrobial nature of the carrier observed, but also the fact that it is stronger for the whole carrier than for the drug itself. This effect was likely due to the relationship occurring between HAp or DCPA and osteoblastic cells, thus confirming the highly osteoconductive nature of these calcium phosphates [69]. The influence of the physicochemical parameters of calcium phosphates on the rate of drug release was also demonstrated. Two types of HAp were subjected to physical sorption in CLD solution; one was freeze-dried initially, and the other was oven-dried at a high temperature. This resulted in changes in the surface morphology, and more drug was released over time from the oven-dried material, where larger agglomerates of grains were observed [70]. In another paper, TCP, HAp, and DCPD were also compared and subjected to CLD modification by a physical sorption process. It was demonstrated that they have a similar potential to be used as DDSs, and that the rate of drug release is affected not only by the porosity, but also by the degree of crystallinity [71]. In each case, the physical sorption process was sufficient to observe CLD release for a minimum of seven days. An interesting solution has been proposed through the use of halloysite, which has applications mainly in catalysis or environmental sciences; however, an increasing number of papers on its biomedical nature have been appearing for some time [72,73]. In this case, the mineral was subjected to CLD modification by intensive stirring and lowering the pressure, which resulted in the migration of drug particles deeper into the material. Experimentally, the carrier was subjected to etching in acetic acid, which did not affect the morphology of the ceramic, but caused an enlargement of its lumen, which resulted in an increased amount of released drug [74]. Table 2 presents a summary of the aforementioned ceramic CLD carriers.

### 4.2. Polymeric Carriers of Clindamycin

**Figure 5:**
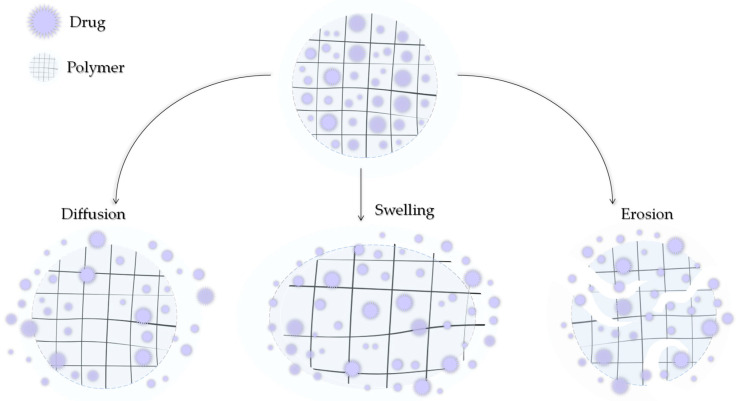
Three main mechanisms of drug release by diffusion, swelling, and erosion.

Modern medicine is unable to avoid polymers. They can be of natural origin or produced synthetically. Depending on the type, structure, and nature, they can exhibit a number of similar but also different, unique properties. Polymers can also be characterized by high chemical resistance or low moisture absorption. Polymers can be lightweight and very strong but also resistant to stretching. An important fact is that they can be easily molded [75,76,77]. By subjecting polymers to appropriate modifications, they might be further adapted to the demanding environment that is the human body. Polymers can be used to produce surgical threads, prostheses, or heart valves and can be applied as DDSs [78,79,80]. A drug can be transported by the polymer carrier in two ways: it can be encapsulated in the structure of the polymer between its chains or bound by a covalent bond directly to the chain or through a suitable ligand [81]. The release of the active ingredient can then occur by the degradation of the polymer, leaching of CLD, or by stimulation by various factors such as the temperature, pH, humidity, and electric or magnetic fields. The three main mechanisms of drug release from the polymer matrix, presented in Figure 5, include hydrogel swelling, material erosion, and diffusion [82].

**Figure 6:**
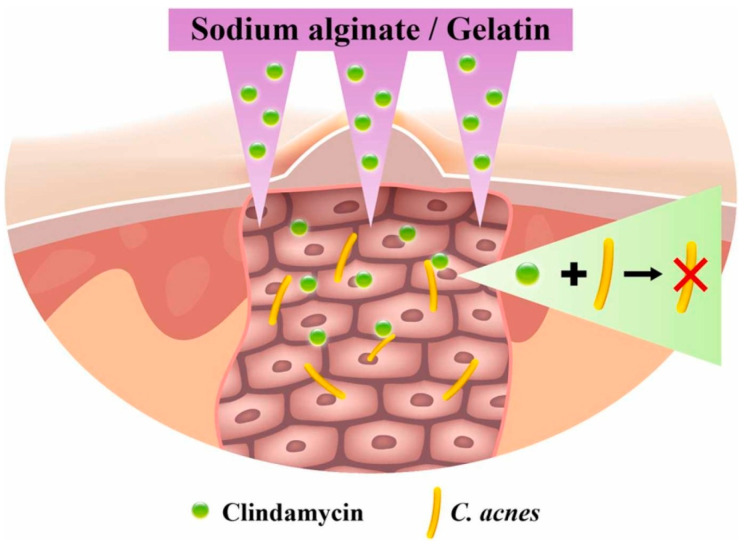
Proposed alginate- and gelatin-based micro-needle solution for the transdermal local delivery of clindamycin. Reprinted with permission from Ref. [92], Elsevier B.V., 2022.

An extremely popular polymer of natural origin used in DDSs, especially in the design of dressing materials, is alginate (ALG). This linear, anionic polysaccharide is derived from brown algae and consists of repeating units of β-1,4-linked d-mannuronic acid (M) and l-guluronic acid (G) in various proportions. It is non-immunogenic, biodegradable, non-toxic, and demonstrates rapid and cell-friendly gelation characteristics [83,84]. Calcium alginate nanoparticles were dispersed in ethanol and a solution of phosphorylated polyalamine containing CLD. The mixture was centrifuged and dried to obtain a precipitate of ALG with the drug. As a result of the centrifugal force generated, the CLD particles were forced deep into the material. Such an arrangement was tested against MG63 cells (line isolated from the bone of a Caucasian, 14-year-old male patient with osteosarcoma [85]) and *S. aureus* strain. Differences were observed due to variations in the pH of the environment in which the analyses were conducted. The system met expectations and demonstrated an antimicrobial effect while not inhibiting the viability of the MG63 cells [86]. An interesting solution was proposed for solid lipid nanoparticles (SLNs) constructed from ALG. They were obtained using an emulsion congealing technique with cold high-pressure homogenization. An oil phase composed of stearic acid and an aqueous phase composed of a CLD solution and a polymer solution were proposed. Analogous SLNs were obtained by replacing ALG with dextrin sulfate. It was observed that it reduced the drug release rate by about 50%, which was probably caused by the higher charge density, lower molecular weight, and lower branching density of the ionic polymer [87]. This confirms that the rate of drug release is dependent on the type of polymer. Other studies proposed a combination of ALG with other biopolymers including chitosan (CHT). The thin films are expected to have applications in skin treatments for acne and for local periodontal therapy. The proportion of individual biopolymers has been proven to affect the physicochemical properties, including the thickness and sorption capacity, and these determine the rate as well as the amount of CLD released [88,89]. CHT is a biocompatible and biodegradable cationic linear polysaccharide composed of *N*-acetyl-d-glucosamine and d-glucosamine, produced by chitin deacetylation [90]. It can be used as a hydrogel itself. Hyperbranched poly((2-(diisopropylamino)ethylmethacrylate)-*b*-(4-formyl-2-methoxyphenyl methacrylate-co-methyl ether poly(ethylene glycol)methacrylate)) nanoparticles were combined with CLD solution by centrifugation and then in a vial with CHT nanoparticles. An injectable gel was thus obtained, from which the drug was released by the pH factor, which caused the imine bonds to break. Not only did it demonstrate antimicrobial properties against *Escherichia coli* and *S. aureus*, but the viability of mouse fibroblast cell line NIH3T cells was higher than 90% [91]. The wide range of manufacturing and material processing methods means that even based on the same starting ingredients, it is ultimately possible to obtain biomaterials for different applications. The combination of gelatin (GE) and ALG can be used to produce materials for bone tissue, soft tissue, and the skin for the treatment of acne vulgaris. Colonization of *Cutibacterium* (*Propionibacterium*) *acnes* is one of the causes of skin deterioration that requires local drug therapy; however, drug particles are usually too large to penetrate the skin. (The *stratum corneum*, the uppermost layer of the skin, acts as a barrier to larger particles.) It has been proposed to create a transdermal patch containing micro-needles composed of ALG and GE (Figure 6). The needles did not pierce the dermis, but penetrated deep enough that the released CLD was able to inhibit the bacterial growth of *C. acnes* in the treated area [92].

Tissue adhesives made of these two polymers have been proposed for the treatment of traumatic wounds, including lacerated skin. Materials containing an antimicrobial agent can protect such a site from SSIs. GE and ALG solutions were mixed with NGC and ECP crosslinking agents. CLD was also added at various concentrations. The addition of the drug was found to improve the adhesive’s bonding strength. When tested against *Staphylococcus albus* and *S. aureus*, complete elimination of the strain was observed within 48 h [93]. As for materials to stimulate bone regeneration processes, ALG was combined with GE nanoparticles. Firstly, the GE nanoparticles were combined with bone morphogenic protein 2 (BMP-2) and CLD in a water bath with homogenization, and then mixed with ALG. BMP-2 itself is a very potent growth factor that induces the rapid differentiation of mesenchymal stem cells into osteoblasts [94]. A functional carrier of the drug and of the active ingredient in the form of BMP-2 protein was obtained and exhibited a dual release of the components over a period of four weeks. The presence of the drug did not interfere with the function of the protein [95]. Alginate hydrogel dressings with pectin and CLD, which were modified with the addition of hyaluronic acid, were developed. The dressings were subjected to in vivo analysis on animal models against male ICR mice, which confirmed the wound healing effect. However, it was observed that the process occurred faster in dressings containing hyaluronic acid. No significant differences were observed in the amount of antibiotic released. The materials themselves were obtained using the solvent casting method [96]. Dressing materials were also obtained by combining a biopolymer with a synthetic polymer such as polyvinyl alcohol (PVA), where solutions of ALG, PVA, and CLD were mixed, subjected to three freezing–thawing cycles, and dried. This simple method yielded flexible carriers that accelerated skin healing [97]. Additional combination with polyvinylpyrrolidone (PVP) and the solvent casting technique resulted in a peel-off mask for acne [98]. The combination with glyceryl monooleate ensures the sustained release of the antibiotic, even after oral administration [99].

The aforementioned PVA can mimic human tissue and is non-toxic, biocompatible, and easy to fabricate [100]. For this reason, this synthetic polymer is often used in tissue engineering alone as well as in combination with other materials. Masks made from an aqueous solution of PVA and CLD obtained by electrospinning or electrospray technique can be used to treat skin wounds [101,102]. PVA mixed with carboxymethylated gum allowed to obtain a patch with very high absorbency, higher than that demonstrated by pure PVA, which resulted in a material for skin oozing wounds, which will be protected from microbial infection by CLD. Electrohydrodynamic atomization was used to obtain the patch from an aqueous solution of the ingredients [103]. Microsponges with ethyl cellulose for the sustained release of CLD have also been proven to be suitable for acne [104]. PVA itself has no use for hard tissue considering its hydrogel nature, which makes it very flexible. Its combination with slightly harder poly(lactic-co-glycolic acid) (PLGA) allowed the production of a DDS for the bone tissue surrounding the teeth. Drug release was observed for as long as three months [105].

PVP was described above in combination with ALG and PVA. Overall, this synthetic polymer appears in many literature reports thanks to its properties. It is not only used to produce carriers and implant materials, but also contact lenses and medical device components [106]. Antimicrobial coatings formulated from PVA and PVP designed to coat catheter surfaces have been proposed to limit the growth of undesirable microorganisms. Thus, the patient would not need to take medication to protect the body, and would be protecting himself from urinary tract infections [107]. PVP was combined with CHT, lactic acid, and CLD to form a carrier ointment for drugs administered vaginally in cases of pregnancies with symptoms of preterm labor, a condition that can be caused by bacterial vaginitis. Clinical evaluation demonstrated that the CLD cream was an effective treatment in this case and enabled permanently maintaining a normal vaginal pH. It also exhibited physicochemical properties similar to those of physiological mucus, thereby proving to be ideal for the vaginal administration of the drug [108].

Another synthetic polymer with potential in biomedical applications is polycaprolactone (PCL). It is a semi-crystalline ester polymer characterized by biodegradability, high strength, and biocompatibility [109]. From solutions of PCL and CLD in dimethylformamide and chloroform, as well as silk fibroin in formic acid, nanofibers were prepared by electrospinning. The addition of fibroin reduced the amount of CLD released [110]. A combination of PCL and GE can be used for both bone and skin applications. The final properties of the carrier will depend on the processing method as well as component ratios. Using electrospinning, dressing materials were produced that released CLD for 6 days. They also demonstrated hydrophilicity and antibacterial properties against a Gram-positive (89%) and a Gram-negative (98%) bacteria [111]. Hard tissue materials were also developed using electrospinning, but the system was further modified by the presence of graphene oxide [112]. Polylactide (PLA) is a polymer with similar characteristics to PCL. It is not soluble in water, so 2,2,2-trifluoroethanol was used as a solvent. It was then mixed with GE and elastin in 1,1,1,3,3,3-hexafluoro- 2-propanol. Patches for skin wound therapy were produced in this way. The antimicrobial effect was confirmed against *S. aureus*, and a biosafety of 87% was validated against human umbilical vein endothelial/vascular endothelium cells. The materials released the CLD and were safe to apply, despite the use of such solvents [113]. By mixing GE with polyethylene glycol dibenzaldehyde using a vortex, a stiff gel was obtained with the formation of imine bonds. The system was crosslinked after 20 s, thus yielding a gel for in situ administration. The addition of CLD did not affect the rate of binding. It was proved that the addition of as little as 2% of the drug was able to kill the entire *S. aureus* colony [114]. Table 3 presents a summary of the aforementioned polymeric CLD carriers.

### 4.3. Composite Carriers of Clindamycin

The basic division of materials into groups includes metals, ceramics, and polymers. Unfortunately, none of them meet all the requirements for implant biomaterials for bone tissue [115]. Metals are often too stiff relative to natural tissue and are associated with the risk of corrosion. This can lead to inflammation and health hazards. Polymers are a broad group of synthetic and natural materials; however, they often have low strength relative to mineralized bone tissue and too high elasticity. Ceramic materials exhibit great brittleness and susceptibility to fracture, but at the same time are often bioactive and can stimulate bone-forming cells to proliferate. For this reason, in order to maintain adequate mechanical, biological, and physicochemical properties, composites, materials formed from more than one phase, are created. Most often, the matrix is a polymer phase providing flexibility, which is reinforced by a dispersed phase in the form of ceramics providing hardness and strength. Developing new composites and combining phases makes it possible to develop biomimetic materials resembling natural tissue, which can create the right microenvironment to promote osteoblast proliferation and osteogenesis. In addition, composites are also good materials for the controlled and sustained release of active substances directly to the site where the therapeutic effect is needed [116,117,118].

In the context of composites for bone tissue regeneration, the most promising combinations are ceramics with polymers. An example is an ALG hydrogel with GE reinforced with a ceramic phase in the form of β-TCP. The materials were obtained by crosslinking based on water solutions. The antimicrobial potential was tested on an ex vivo human bone infection organ model [119,120]. The electrospinning method made it possible to obtain tough composite layers based on a mixture of GE, a natural polymer, and PCL, a synthetic polymer. The surface was modified with graphene oxide, which later attached DCPD through ionic interactions. The CLD was encapsulated in a polymer phase. Nanofibers obtained in this way demonstrated good biocompatibility with human osteosarcoma cells as well as no cell toxicity. Slow and controlled release of CLD but also DCPD was observed in vitro [112]. Another composite combination, whose polymer phase consists of a biological component and a synthetic one, is CHT with PLGA. It was reinforced with HAp in the form of suspended nanocrystals. CLD was combined with the polymer phase, and the composite was obtained by freeze-drying the mixture. The final form analyzed was films. The release of the antibiotic occurred by the slow degradation of CHT/PLGA and by swelling [121]. Despite the degradability exhibited by CHT, sustained release of CLD was observed for three weeks for composites based on only this biopolymer and nano-HAp. However, it was observed that with the addition of CHT, the antimicrobial efficacy of this DDS against *S. aureus* decreased. The reduced proliferation of MC3T3-E1 osteoblastic cells as well as mitochondrial dehydrogenase activity was also observed, which may suggest that CHT, despite its rather good biocompatibility properties, is not entirely suitable for such applications [122]. Of all biocompatible ceramics, it is HAp that is most commonly used in biomedical applications. Combined with PVP and betaine, again a combination of synthetic and biological components, it was used for the creation of a platform in the form of a flexible composite obtained by photocrosslinking, which exhibited good physicochemical properties. Sustained release of CLD was observed for 7 days, which proved that the increased ceramics phase reduced the amount of the drug released in vitro. The UV crosslinking process did not lead to drug degradation [123]. PVP was also used to develop bone cement composites modified with CLD, triclosan, or gentamicin as DDS materials for arthroplasty. The drugs were bonded to the cements, and PVP was intended to increase their leaching from the cement. However, for CLD and gentamicin, no difference was observed between the amount of drug released from the cement alone and from the cement with the polymer. This difference was observed only for triclosan and may be due to the difference in the chemical structure and origin of the three drugs, because triclosan comes from the chlorinated phenol group [124,125].

PLA is widely used in biomedicine to print bone defects; however, its melting point is high enough, so active compounds could degrade [126]. For this reason, to use it as a DDS, a printing technique cannot be applied; however, such a polymer can be dissolved in suitable solvents and processed appropriately. PLA was dissolved in acetonitrile, and CLD and HAp were then added to the solution. Ultrasonic radiation was used to disintegrate the agglomerates, and finally, microcapsules were obtained. The ceramic phase was of natural origin, from crocodile bone. The loading efficiency of the CLD composite was demonstrated to increase with the proportion of HAp [127]. PLGA was also dissolved in acetonitrile. As before, CLD solution was added to the polymer; ultrasound was used. In this case, HAp and other calcium phosphate powders were also modified with CLD. Antimicrobial properties against *S. aureus* were demonstrated, and biosafety against osteoblast-like cells was confirmed despite the use of acetonitrile, a solvent that is toxic to the organism [128].

**Figure 7:**
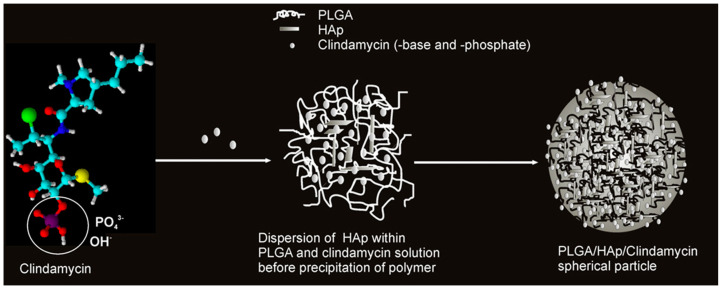
Proposed mechanism for the formation of spherical CLD-based poly(lactic-co-glycolic acid)/hydroxyapatite (PLGA/HAp) core–shell nanoparticles. Reprinted with permission from ref. [129], Elsevier B.V., 2011.

Acetone is also used as a solvent for PLGA. Therefore, PLGA in acetone was combined with a solution of CLD and HAp powder. Nanospheres capable of releasing the drug, the amount of which was determined by the content of the ceramic phase, were obtained. A schematic of this carrier formulation mechanism is presented in Figure 7. The nanoparticles were obtained by using ultrasound. The drug and ceramic grains were encapsulated in a polymer network. It was confirmed that CLD influenced the degradation of the polymer matrix, which also affected the amount of released antibiotic. A high level of cytocompatibility was observed against mouse L929 and human lung MRC-5 fibroblasts [129,130,131,132]. Not only popular bioactive ceramics in the form of TCP or HAp can be used as a reinforcing phase in the composite as the literature mentions the use of, for example, montmorillonite clay or bentonite. The first one is often used as an ingredient in skin care masks for its purifying and absorbing properties [133]. It was mixed with an aqueous solution of PVA and CLD. A crosslinking method by cyclic freeze–thaw was used. The resulting wound healing dressings exhibited antimicrobial properties due to the presence of the drug and the clay [134]. Bentonite was combined with PVP and carboxymethylcellulose (CMC) to obtain a DDS [135]. In order to ensure the flexibility of composites, they were enriched with the addition of xanthan gum and guar gum. This non-obvious combination resulted in obtaining a CLD carrier using the Petri dish casting method [136]. CMC is a derivative of cellulose, and its main area of application is in the food industry. It belongs to the group of emulsifiers or thickening agents and is labeled as E466 [137]. Its combination with halloysite, previously modified with CLD, and keratin gave nanocomposites with antimicrobial properties for skin wound healing. Drug release occurred according to the Fickian diffusion mechanism [138]. The last non-obvious combination discussed in this review is a coating composed of CMC and ALG. Naturally, drug molecules were encapsulated in the polymer network. The coating was applied by electrospinning to TiAlV and 316LVM sheets. A sandwich composite was thus obtained, where the layers of the individual materials were arranged on top of each other, rather than suspended in each other as in the previously discussed composites. CLD was released into a fluid simulating a biological environment, and the release occurred by the degradation process of the polymer phase [139]. Table 4 presents a summary of the aforementioned composites for CLD delivery.

## 5. Conclusions and Future Challenges

New biomaterials can indeed transform modern medicine and therapy, providing relief to suffering and needy patients. Smart biomaterials are essential for this purpose. In the current situation, as demonstrated above, newly emerging ceramic, polymeric, and composite materials are exhibiting many advantages for their application as controlled DDSs. Materials from each group, synthetic as well as natural, are all being used in the design of new DDSs. As demonstrated in this review, depending on the purpose and target site of the implantation or application of the biomaterial, different phases are used. In the case of bone tissue, ceramics and ceramic–polymer composites are mainly investigated. It was observed that the most common ceramics of choice were bioactive calcium phosphates, such as HAp, which stimulate the activity of bone-forming cells. In the case of composites, polymers in which ceramics in the form of fine grains were homogeneously suspended in the material accounted for the greatest share. Mostly, these were synthetic polymers like PLGA or PVP. However, in the case of soft tissue or skin, biopolymers of natural origin are most often used. The most reported are ALG and GE. Physical sorption in a solution or mixing with an aqueous CLD solution was the most common method for the modification of individual phases with the drug.

However, despite enormous advances in targeted therapy and the controlled delivery of active substances, there are still many issues to be solved and improved. Unfortunately, the release of the drug often occurs too fast and does not fit into the idea of controlled sustained release. This is likely the reason why the simple phenomenon of sorption and not chemical bonding is used. The challenge is to create a carrier that will release the drug at an appropriate rate, while exhibiting a range of characteristics and properties ideally similar to those of the tissue being replaced. At present, it seems that one of the main goals in relation to DDS biomaterials is to develop a material with adequate mechanical parameters while maintaining a sustained release of the active substance. Biomimeticism is also being considered to ensure that the new materials mimic natural tissues as closely as possible. Targeted therapy represents the future of medicine by providing faster and more effective treatment directly at the site that requires it. Thus, further development in this field is essential and important for the life of society and improving the quality of life of those in need.

## Figures and Tables

**Table 1:** Advantages and disadvantages of using clindamycin.

Advantages	Disadvantages
Can be administered both systemically and locally; Diet has no effect on efficacy; Active against most aerobic and anaerobic Gram-positive bacteria; Increases intracellular killing of susceptible organisms, reduces bacterial adhesion to host cells; Has a positive effect on the overall recovery outcome, as it does not block pro-angiogenic activity.	Side effects such as allergic reactions, colitis, nausea, vomiting, and diarrhea; Can cause esophagitis, taste disorders, and changes in hematological parameters; Poor permeability of the outer cell envelope, thus not working well against aerobic Gram-negative bacilli.

**Table 2:** Ceramic carriers of clindamycin with indication of material type and applications.

Material	Applications	Refs.
ACP	NPs as intrinsic inorganic antimicrobials	[68]
HAp	NPs as intrinsic inorganic antimicrobials DDSs in form of powders and disks for hard tissue engineering	[68,69,71]
DCPA	NPs powders for hard tissue engineering	[69]
DCPD	DDSs for hard tissue engineering	[71]
TCP	DDSs for hard tissue engineering	[71]
Halloysite	Nanotubes with antibacterial properties	[74]

ACP = amorphous calcium phosphate, HAp = hydroxyapatite, DCPA = dicalcium phosphate anhydrous, DCPD = dicalcium phosphate dihydrate, DDSs = drug delivery systems, NPs = nanoparticles, TCP = calcium triphosphate.

**Table 3:** Polymeric carriers of clindamycin with indication of material type and applications.

Material	Application	Refs.
ALG	Materials for osteomyelitis treatment	[79,80]
ALG, CHT	Skin treatments for acne and for local periodontal therapy	[81,82]
CHT	Soft hydrogel, in form of injectable gel	[84]
ALG, GE	Transdermal patch containing micro-needles for the treatment of acne vulgaris	[85]
ALG, GE	Materials for the treatment of traumatic wounds, lacerated skin	[86]
ALG, GE, BMP-2	Drug delivery systems for hard tissue engineering	[87]
ALG, pectin, hyaluronic acid	Dressing materials for wound healing	[89]
ALG, PVA	Dressing materials for wound healing	[90]
ALG, PVA, PVP	Hydrogel peel-off mask for acne	[91]
PVA	Hydrogel mask to treat skin wounds	[94,95]
PVA, carboxymethylated gum	Material for skin oozing wounds	[96]
PVA, ethyl cellulose	Microsponge for acne	[97]
PVA, PLGA	Bone tissue surrounding the teeth	[98]
PVA, PVP	Materials for urinary tract infections	[100]
PVP, CHT, lactid acid	Ointment for drugs administered vaginally	[101]
PCL, fibroin	Biocompatible nanofibers with core–shell structures for multiple applications as tissue engineering scaffolds	[103]
PCL, GE	Bone and skin applications	[104,105]
PLA, GE, elastin	Patches for skin wound therapy	[106]
GE, polyethylene glycol	Self-healing, injectable hydrogels	[107]

ALG = alginate, BMP-2 = bone morphogenic protein 2, CHT = chitosan, GE = gelatin, PCL = polycaprolactone, PLA = polylactide, PLGA = poly(lactic-co-glycolic acid), PVA = polyvinyl alcohol, PVP = polyvinylpyrrolidone.

**Table 4:** Composite materials for delivery of clindamycin with indication of material type and applications.

Material	Application	Refs.
PCL, GE, DCPD, graphene oxide	Microcapsules for local drug delivery and for bone regeneration	[112]
PLA, GE, TCP	Composites for bone regeneration	[113]
GE, PCL, DCPD	Nanofibers for application in bone tissue engineering	[114]
PLGA, CHT, HAp	Composite films with antimicrobial properties	[115]
CHT, HAp	Biodegradable materials for bone tissue	[116]
PVP, betaine, HAp	Elastic materials for hard tissue replacement	[117]
PVP, ceramic cements	Materials for treatment in two-stage arthroplasty revisions	[119]
PLGA, HAp	Nanospheres capable of releasing the drug locally	[121,122,123,124,125]
PVA, montmorillonite	Wound healing dressings	[127]
PVP, CMC, bentonite	Biodegradable hydrogel films for biomedical application	[128]
CMC, keratin, halloysite	Nanocomposites with antimicrobial properties for skin wound	[131]
CMC, ALG, TiAlV/316LVM	Sandwich composite for bone tissue	[132]

ALG = alginate, GE = gelatin, CHT = chitosan, CMC = carboxymethylcellulose, DCPD = dicalcium phosphate dehydrate, HAp = hydroxyapatite, PCL = polycaprolactone, PLA = polylactide, PLGA = poly(lactic-co-glycolic acid), PVA = polyvinyl alcohol, PVP = polyvinylpyrrolidone, TCP = calcium triphosphate.

## Data Availability

Not applicable.

## References

[1] Ghanem A.M. (2024). Recent advances in transdermal drug delivery systems: A review. Int. J. Appl. Pharm..

[2] Singh S., Pandey V.K., Tewari R.P., Agarwal V. (2011). Nanoparticle based drug delivery system: Advantages and applications. Ind. J. Sci. Technol..

[3] Drug Delivery Systems Market Size, Share & COVID-19 Impact Analysis, by Type (Inhalation, Transdermal, Injectable, and Others), by Device Type (Conventional and Advaced), by Distribution Channel (Hospital, Pharmacies, Retail Pharmacies, and Others). https://www.fortunebusinessinsights.com/drug-delivery-systems-market-103070.

[4] Adepu S., Ramakrishna S. (2021). Controlled drug delivery systems: Current status and future directions. Molecules.

[5] Dash T.R., Verma P. (2013). Matrix tablets: An approach towards oral extended release drug delivery. Int. J. Pharma Res. Rev..

[6] Pinto J.F. (2010). Site-specific drug delivery systems within the gastro-intestinal tract: From the mouth to the colon. Int. J. Pharm..

[7] Reddy D., Pillay V., Choonara Y.E., Du Toit L.C. (2009). Rapidly disintegrating oramucosal drug delivery technologies Rapidly disintegrating oramucosal drug delivery technologies. Pharm. Develop. Technol..

[8] Anderson G.D., Saneto R.P. (2012). Current oral and non-oral routes of antiepileptic drug delivery. Adv. Drug Deliv. Rev..

[9] Matsuda Y., Konno Y., Hashimoto T., Nagai M., Taguchi T., Satsukawa M., Yamashita S. (2015). Quantitative assessment of intestinal first-pass metabolism of oral drugs using portal-vein cannulated rats. Pharm. Res..

[10] Riviere J.E., Papich M.G. (2001). Potential and problems of developing transdermal patches for veterinary applications. Adv. Drug Deliv. Rev..

[11] Escobar-Chavez J.J., Diaz-Torres R., Rodriguez-Cruz I.M., Dominguez-Delgado C.L., Sampere-Morales R., Angeles-Anguiano E., Melgoza-Contreras L.M. (2012). Nanocarriers for transdermal drug delivery. Res. Rep. Transdermal Drug Deliv..

[12] Păduraru D.N., Niculescu A.-G., Bolocan A., Andronic O., Grumezescu A.M., Bîrlă R. (2022). An updated overview of cyclodextrin-based drug delivery systems for cancer therapy. Pharmaceutics.

[13] Spoială A., Ilie C.-I., Motelica L., Ficai D., Semenescu A., Oprea O.-C., Ficai A. (2023). Smart magnetic drug delivery systems for the treatment of cancer. Nanomaterials.

[14] Rizwan M., Yahya R., Hassan A., Yar M., Azzahari A.D., Selvanathan V., Sonsudin F., Abouloula C.N. (2017). pH Sensitive hydrogels in drug delivery: Brief history, properties, swelling, and release mechanism, material selection and applications. Polymers.

[15] Ayran M., Karabulut H., Deniz K.I., Akcanli G.C., Ulag S., Croitoru A.-M., Tihăuan B.-M., Sahin A., Ficai D., Gunduz O. (2023). Electrically triggered quercetin release from polycaprolactone/bismuth ferrite microfibrous scaffold for skeletal muscle tissue. Pharmaceutics.

[16] Silindir Gunay M., Yekta Ozer A., Chalon S. (2015). Drug delivery systems for imaging and therapy of Parkinson’s disease. Curr. Neuropharm..

[17] Huang X., Ma Y., Li Y., Han F., Lin W. (2021). Targeted drug delivery systems for kidney diseases. Front. Bioeng. Biotechnol..

[18] Paolini M.S., Fenton O.S., Bhattacharya C., Andresen J.L., Langer R.S. (2019). Polymers for extended-release administration. Biomed. Microdevices.

[19] Kleiner L.W., Wright J.C., Wang Y. (2014). Evolution of implantable and insertable drug delivery systems. J. Control. Release.

[20] Van Tran T.T., Tayara H., Chong K.T. (2023). Artificial intelligence in drug metabolism and excretion prediction: Recent advances, challenges, and future perspectives. Pharmaceutics.

[21] Hardenia A., Maheshwari N., Hardenia S.S., Dwivedi S.K., Maheshwari R., Tekade R.K., Tekade R.K. (2019). Scientific rationale for designing controlled drug delivery systems. Basic Fundamentals of Drug Delivery—Advances in Pharmaceutical Product Development and Research.

[22] Paarakh M.P., Jose P.A.N.I., Setty C.M., Peter G.V. (2019). Release Kinetics—Concepts and applications. Int. J. Pharm. Res. Technol..

[23] Wiseman J.T., Fernandes-Taylor S., Barnes M.L., Saunders R.S., Saha S., Havlena J., Rathouz P.J., Craig Kent K. (2015). Predictors of surgical site infection after hospital discharge in patients undergoing major vascular surgery. J. Vasc. Surg..

[24] Ibrahimi O.A., Sharon V., Eisen D.B. (2011). Surgical-Site infections and routes of bacterial transfer: Which ones are most plausible?. Dermatol. Surg..

[25] Ali K.M., Al-Jaff B.M.A. (2021). Source and antibiotic susceptibility of Gram-negative bacteria causing superficial incisional surgical site infections. Int. J. Surg. Open.

[26] Daeschlein G., Napp M., Layer F., von Podewils S., Haase H., Spitzmueller R., Assadian O., Kasch R., Werner G., Jünger M. (2015). Antimicrobial efficacy of preoperative skin antisepsis and clonal relationship to postantiseptic skin-and-wound flora in patients undergoing clean orthopedic surgery. Eur. J. Clin. Microbiol. Inf. Dis..

[27] Brady R.A., Leid J.G., Costerton J.W., Shirtliff M.E. (2006). Osteomyelitis: Clinical overview and mechanisms of infection persistence. Clin. Microbiol. Newslet..

[28] Rimashevskiy D.V., Akhtyamov I.F., Fedulichev P.N., Zaalan W., Ustazov K.A., Basith A., Moldakulov J.M., Zinoviev M.P. (2021). Pathogenetic features of chronic osteomyelitis treatment. Genij Ortopedii.

[29] Lam M., Hu A., Fleming P., Lynde C.W. (2022). The impact of acne treatment on skin bacterial microbiota: A systematic review. J. Cut. Med. Surg..

[30] Wang C., Huttner B.D., Magrini N., Cheng Y., Tong J., Li S., Wan C., Zhu Q., Zhao S., Zhuo Z. (2020). Pediatric antibiotic prescribing in China according to the 2019 World Health Organization access, watch, and reserve (AWaRe) antibiotic categories. J. Pediatr..

[31] Vekemans J., Hasso-Agopsowicz M., Kang G., Hausdorff W.P., Fiore A., Tayler E., Klemm E.J., Laxminarayan R., Srikantiah P., Friede M. (2021). Leveraging Vaccines to reduce antibiotic use and prevent antimicrobial resistance: A World Health Organization action framework. Clin. Inf. Dis..

[32] Luchian I., Goriuc A., Martu M.A., Covasa M. (2021). Clindamycin as an alternative option in optimizing periodontal therapy. Antibiotics.

[33] Álvarez L.A., Van de Sijpe G., Desmet S., Metsemakers W.J., Spriet I., Allegaert K., Rozenski J. (2022). Ways to Improve insights into clindamycin pharmacology and pharmacokinetics tailored to practice. Antibiotics.

[34] Chaiwarit T., Rachtanapun P., Kantrong N., Jantrawut P. (2020). Preparation of clindamycin hydrochloride loaded de-esterified low-methoxyl mango peel pectin film used as a topical drug delivery system. Polymers.

[35] Egle K., Skadins I., Grava A., Micko L., Dubniks V., Salma I., Dubnika A. (2022). Injectable platelet-rich fibrin as a drug carrier increases the antibacterial susceptibility of antibiotic—clindamycin phosphate. Int. J. Mol. Sci..

[36] Spížek J., Řezanka T. (2017). Lincosamides: Chemical structure, biosynthesis, mechanism of action, resistance, and applications. Biochem. Pharmacol..

[37] Assefa M. (2022). Inducible clindamycin-resistant *Staphylococcus aureus* strains in Africa: A systematic review. Int. J. Microbiol..

[38] Cherazard R., Epstein M., Doan T.-L., Salim T., Bharti S., Smith M.A. (2017). Antimicrobial resistant *Streptococcus pneumoniae*: Prevalence, mechanisms, and clinical implications. Am. J. Ther..

[39] Cheung G.Y.C., Bae J.S., Otto M. (2021). Pathogenicity and virulence of *Staphylococcus aureus*. Virulence.

[40] Mohamed N., Wang M.Y., Le Hue J.-C., Liljenqvist U., Scully I.L., Baber J., Begier E., Jansen K.U., Gurtman A., Anderson A.S. (2017). Vaccine development to prevent *Staphylococcus aureus* surgical-site infections. Brit. J. Surg..

[41] Li L., Ma J., Yu Z., Li M., Zhang W., Sun H. (2023). Epidemiological characteristics and antibiotic resistance mechanisms of *Streptococcus pneumoniae*: An updated review. Microbiol. Res..

[42] Wade K.C., Benjamin D.K., Remington J.S., Klein J.O., Wilson C.B., Nizet V., Maldonado Y.A. (2011). Clinical pharmacology of anti-infective drugs. Infectious Diseases of the Fetus and Newborn.

[43] Rezahosseini O., Roed C., Holler J.G., Frimodt-Møller N. (2023). Adjunctive antibiotic therapy with clindamycin or linezolid in patients with group A *Streptococcus* (GAS) *meningitis*. Inf. Dis..

[44] Arteagoitia I., Sánchez F.R., Figueras A., Arroyo-Lamas N. (2022). Is clindamycin effective in preventing infectious complications after oral surgery? Systematic review and meta-analysis of randomized controlled trials. Clin. Oral Investig..

[45] Peeters A., Putzeys G., Thorrez L. (2019). Current insights in the application of bone grafts for local antibiotic delivery in bone reconstruction surgery. J. Bone Jt. Infect..

[46] Allegaert K., Muller A.E., Russo F., Schoenmakers S., Deprest J., Koch B.C.P. (2020). Pregnancy-related pharmacokinetics and antimicrobial prophylaxis during fetal surgery, cefazolin and clindamycin as examples. Prenat. Diagn..

[47] Eda T., Okada M., Ogura R., Tsukamoto Y., Kanemaru Y., Watanabe J., On J., Aoki H., Oishi M., Takei N. (2022). Novel repositioning therapy for drug-resistant glioblastoma: In vivo validation study of clindamycin treatment targeting the mTOR pathway and combination therapy with temozolomide. Cancers.

[48] Yang S.H., Lee M.G. (2007). Dose-independent pharmacokinetics of clindamycin after intravenous and oral administration to rats: Contribution of gastric first-pass effect to low bioavailability. Int. J. Pharm..

[49] Lemaire S., Van Bambeke F., Pierard D., Appelbaum P.C., Tulkens P.M. (2011). Activity of fusidic acid against extracellular and intracellular *Staphylococcus aureus*: Influence of ph and comparison with linezolid and clindamycin. Clin. Inf. Dis..

[50] Hua S. (2020). Advances in oral drug delivery for regional targeting in the gastrointestinal tract—Influence of physiological, pathophysiological and pharmaceutical factors. Front. Pharmacol..

[51] Vellonen K.S., Soini E.M., Del Amo E.M., Urtti A. (2016). Prediction of ocular drug distribution from systemic blood circulation. Mol. Pharm..

[52] Thornhill M.H., Dayer M.J., Durkin M.J., Lockhart P.B., Baddour L.M. (2019). Risk of adverse reactions to oral antibiotics prescribed by dentists. J. Dent. Res..

[53] Dubey N., Xu J., Zhang Z., Nör J.E., Bottino M.C. (2019). Comparative evaluation of the cytotoxic and angiogenic effects of minocycline and clindamycin: An in vitro study. J. Endod..

[54] Eliaz N., Metoki N. (2017). Calcium phosphate bioceramics: A review of their history, structure, properties, coating technologies and biomedical applications. Materials.

[55] Canillas M., Pena P., De Aza A.H., Rodríguez M.A. (2017). Calcium phosphates for biomedical applications. Bol. Soc. Esp. Ceram. Vidr..

[56] Lara-Ochoa S., Ortega-Lara W., Guerrero-Beltrán C.E. (2021). Hydroxyapatite nanoparticles in drug delivery: Physicochemistry and applications. Pharmaceutics.

[57] Harb S.V., Bassous N.J., de Souza T.A.C., Trentin A., Pulcinelli S.H., Santilli C.V., Webster T.J., Lobo A.O., Hammer P. (2020). Hydroxyapatite and β-TCP modified PMMA-TiO_2_ and PMMA-ZrO_2_ coatings for bioactive corrosion protection of Ti_6_Al_4_V implants. Mat. Sci. Eng. C.

[58] Shalini B., Kumar A.R. (2019). A comparative study of hydroxyapatite (Ca_10_(PO_4_)_6_(OH)_2_) using sol-gel and co-precipitation methods for biomedical applications. J. Indian Chem. Soc..

[59] Damerau J.M., Bierbaum S., Wiedemeier D., Korn P., Smeets R., Jenny G., Nadalini J., Stadlinger B. (2022). A systematic review on the effect of inorganic surface coatings in large animal models and meta-analysis on tricalcium phosphate and hydroxyapatite on periimplant bone formation. J. Biomed. Mat. Res. B.

[60] Jeong J., Kim J.H., Shim J.H., Hwang N.S., Heo C.Y. (2019). Bioactive calcium phosphate materials and applications in bone regeneration. Biomater. Res..

[61] Wagner M., Hess T., Zakowiecki D. (2022). Studies on the pH-dependent solubility of various grades of calcium phosphate-based pharmaceutical excipients. J. Pharm. Sci..

[62] El Hazzat M., El Hamidi A., Halim M., Arsalane S. (2021). Complex evolution of phase during the thermal investigation of Brushite-type calcium phosphate CaHPO_4_·2H_2_O. Materialia.

[63] Gbureck U., Vorndran E., Barralet J.E. (2008). Modeling vancomycin release kinetics from microporous calcium phosphate ceramics comparing static and dynamic immersion conditions. Acta Biomat..

[64] Zamoume O., Thibault S., Regnié G., Mecherri M.O., Fiallo M., Sharrock P. (2011). Macroporous calcium phosphate ceramic implants for sustained drug delivery. Mat. Sci. Eng. C.

[65] Mo X., Zhang D., Liu K., Zhao X., Li X., Wang W. (2023). Nano-hydroxyapatite composite scaffolds loaded with bioactive factors and drugs for bone tissue engineering. Int. J. Mol. Sci..

[66] Wang L., Hou X., Feng L., Zhou Y., Liu X., Tian C. (2022). Drug delivery properties of three—Dimensional ordered macroporous zinc—Doped hydroxyapatite. J. Mat. Res..

[67] Munir M.U., Salman S., Javed I., Bukhari S.N.A., Ahmad N., Shad N.A., Aziz F. (2021). Nano-hydroxyapatite as a delivery system: Overview and advancements. Artif. Cells Nanomed Biotechnol..

[68] Wu V.M., Tang S., Uskoković V. (2018). Calcium phosphate nanoparticles as intrinsic inorganic antimicrobials: The antibacterial effect. ACS Appl. Mater. Interfaces.

[69] Uskoković V., Desai T.A. (2014). Simultaneous bactericidal and osteogenic effect of nanoparticulate calcium phosphate powders loaded with clindamycin on osteoblasts infected with *Staphylococcus aureus*. Mat. Sci. Eng. C.

[70] Niziołek K., Słota D., Sadlik J., Łachut E., Florkiewicz W., Sobczak-Kupiec A. (2023). Influence of drying technique on physicochemical properties of synthetic hydroxyapatite and its potential use as a drug carrier. Materials.

[71] Słota D., Piętak K., Florkiewicz W., Jampílek J., Tomala A., Urbaniak M.M., Tomaszewska A., Rudnicka K., Sobczak-Kupiec A. (2023). Clindamycin-loaded nanosized calcium phosphates powders as a carrier of active substances. Nanomaterials.

[72] Massaro M., Noto R., Riela S. (2020). Past, present and future perspectives on halloysite clay minerals. Molecules.

[73] Danyliuk N., Tomaszewska J., Tatarchuk T. (2020). Halloysite nanotubes and halloysite-based composites for environmental and biomedical applications. J. Mol. Liq..

[74] Machowska A., Klara J., Ledwójcik G., Wójcik K., Dulińska-Litewka J., Karewicz A. (2022). Clindamycin-loaded halloysite nanotubes as the antibacterial component of composite hydrogel for bone repair. Polymers.

[75] Cywar R.M., Rorrer N.A., Hoyt C.B., Beckham G.T., Chen E.Y.-X. (2022). Bio-based polymers with performance-advantaged properties. Nat. Rev. Mat..

[76] Zhao J., Wang G., Wang C., Park C.B. (2020). Ultra-lightweight, super thermal-insulation and strong PP/CNT microcellular foams. Compos. Sci. Technol..

[77] Kraft U., Molina-Lopez F., Son D., Bao Z., Murmann B. (2020). Ink development and printing of conducting polymers for intrinsically stretchable interconnects and circuits. Adv. Electron. Mater..

[78] Hart L.F., Hertzog J.E., Rauscher P.M., Rawe B.W., Tranquilli M.M., Rowan S.J. (2021). Material properties and applications of mechanically interlocked polymers. Nat. Rev. Mat..

[79] Rezvova M.A., Klyshnikov K.Y., Gritskevich A.A., Ovcharenko E.A. (2023). Polymeric heart valves will displace mechanical and tissue heart valves: A new era for the medical devices. Int. J. Mol. Sci..

[80] Bărăian A.-I., Iacob B.-C., Bodoki A.E., Bodoki E. (2022). In vivo applications of molecularly imprinted polymers for drug delivery: A pharmaceutical perspective. Int. J. Mol. Sci..

[81] Hartl N., Adams F., Merkel O.M. (2021). From Adsorption to Covalent Bonding: Apolipoprotein E Functionalization of polymeric nanoparticles for drug delivery across the blood–brain barrier. Adv. Ther..

[82] Priya James H., John R., Alex A., Anoop K.R. (2014). Smart polymers for the controlled delivery of drugs—A concise overview. Acta Pharm. Sin. B.

[83] Gao Q., Kim B.-S., Gao G. (2021). Advanced strategies for 3D bioprinting of tissue and organ analogs using alginate hydrogel bioinks. Mar. Drugs.

[84] Zhang M., Zhao X. (2020). Alginate hydrogel dressings for advanced wound management. Int. J. Biol. Macromol..

[85] Wagener N., Di Fazio P., Böker K.O., Matziolis G. (2022). Osteogenic effect of pregabalin in human primary mesenchymal stem cells, osteoblasts and osteosarcoma cells. Life.

[86] Gowri M., Latha N., Suganya K., Rajan M. (2021). Calcium alginate nanoparticle crosslinked phosphorylated polyallylamine to the controlled release of clindamycin for osteomyelitis treatment controlled release of clindamycin for osteomyelitis treatment. Drug Dev. Ind. Pharm..

[87] Abbaspour M., Makhmalzadeh B.S., Arastoo Z., Jahangiri A., Shiralipour R. (2013). Effect of anionic polymers on drug loading and release from clindamycin phosphate solid lipid nanoparticles. Trop. J. Pharm. Res..

[88] Kilicarslan M., Ilhan M., Inal O., Orhan K. (2018). Preparation and evaluation of clindamycin phosphate loaded chitosan/alginate polyelectrolyte complex film as mucoadhesive drug delivery system for periodontal therapy. Eur. J. Pharm. Sci..

[89] Zanganeh S.M., Tahvildari K., Nozari M. (2023). Preparation and characterization of chitosan-alginate biopolymer loaded by clindamycin phosphate as an effective drug delivery system for the treatment of acne. Polym. Bull./Res. Sq..

[90] Do N.H.N., Truong Q.T., Le P.K., Ha A.C. (2022). Recent developments in chitosan hydrogels carrying natural bioactive compounds. Carbohydr. Polym..

[91] Wei S., Liu X., Zhou J., Zhang J., Dong A., Huang P., Wang W., Deng L. (2020). Dual-crosslinked nanocomposite hydrogels based on quaternized chitosan and clindamycin-loaded hyperbranched nanoparticles for potential antibacterial applications. Int. J. Biol. Macromol..

[92] Tiraton T., Suwantong O., Chuysinuan P., Ekabutr P., Niamlang P., Khampieng T., Supaphol P. (2022). Biodegradable microneedle fabricated from sodium alginate-gelatin for transdermal delivery of clindamycin. Mater. Today Commun..

[93] Foox M., Raz-Pasteur A., Berdicevsky I., Krivoy N., Zilberman M. (2014). In vitro microbial inhibition, bonding strength, and cellular response to novel gelatin-alginate antibiotic-releasing soft tissue adhesives. Polym. Adv. Technol..

[94] Dirzu N., Lucaciu O., Dirzu D.S., Soritau O., Cenariu D., Crisan B., Tefas L., Campian R.S. (2022). BMP-2 delivery through liposomes in bone regeneration. Appl. Sci..

[95] Schrade S., Ritschl L., Süss R., Schilling P., Seidenstuecker M. (2022). Gelatin Nanoparticles for targeted dual drug release out of alginate-di-aldehyde-gelatin gels. Gels.

[96] Hasan N., Cao J., Lee J., Kim H., Wook J. (2021). Development of clindamycin—Loaded alginate/pectin/hyaluronic acid composite hydrogel film for the treatment of MRSA—infected wounds. J. Pharm. Investig..

[97] Kim J.O., Choi J.Y., Park J.K., Kim J.H., Jin S.G., Chang S.W., Li D.X., Hwang M.R., Woo J.S., Kim J.A. (2008). Development of clindamycin-loaded wound dressing with polyvinyl alcohol and sodium alginate. Biol. Pharm. Bull..

[98] Morakul B., Wongrakpanich A., Teeranachaidekul V., Washiradathsathien K., Gamolvate A. (2023). Clindamycin peel-off mask film, an effective formulation for *C. acnes* treatment: Characterization and microbiological activity. Indonesian J. Pharm..

[99] Mohamed A.I., Ahmed O.A., Amin S., Elkadi O.A., Kassem M.A. (2015). In-vivo evaluation of clindamycin release from glyceryl monooleate-alginate microspheres by NIR spectroscopy. Int. J. Pharm..

[100] Jalageri M.B., Mohan Kumar G.C. (2022). Hydroxyapatite reinforced polyvinyl alcohol/polyvinyl pyrrolidone based hydrogel for cartilage replacement. Gels.

[101] Nadem S., Ziyadi H., Hekmati M., Baghali M. (2020). Cross—linked poly(vinyl alcohol) nanofibers as drug carrier of clindamycin. Polym. Bull..

[102] Mandegari M., Ghasemi-Mobarakeh L., Varshosaz J. (2022). Fabrication and characterization of a novel wound dressing with clindamycin loaded PVA nanoparticles for acne treatment. Fiber. Polym..

[103] Sangnim T., Limmatvapirat S., Nunthanid J., Sriamornsak P., Sittikijyothin W., Wannachaiyasit S., Huanbutta K. (2018). Design and characterization of clindamycin-loaded nanofiber patches composed of polyvinyl alcohol and tamarind seed gum and fabricated by electrohydrodynamic atomization. Asian J. Pharm. Sci..

[104] Khattab A., Nattouf A. (2021). Optimization of entrapment efficiency and release of clindamycin in microsponge based gel. Sci. Rep..

[105] Ilhan M., Kilicarslan M., Orhan K. (2022). Effect of process variables on in vitro characteristics of clindamycin phosphate loaded PLGA nanoparticles in dental bone regeneration and 3D characterization studies using nano-CT. J. Drug Deliv. Technol..

[106] Kurakula M., Rao G.S.N.K. (2020). Pharmaceutical assessment of polyvinylpyrrolidone (PVP): As excipient from conventional to controlled delivery systems with a spotlight on COVID-19 inhibition. J. Drug Deliv. Technol..

[107] Borowska M., Glinka M., Filipowicz N., Terebieniec A., Szarlej P. (2019). Polymer biodegradable coatings as active substance release systems for urological applications. Monatsh. Chem..

[108] Hirnle L., Heimrath J., Woytoń J., Kłósek A., Hirnle G., Małolepsza-Jarmołowska K. (2002). Application of 2% clindamycin cream in the treatment of bacterial vaginosis and valuation of methylcellulose gel containing the complex of chitosan F and PVP k-90 with lactic acid as carrier for intravaginally adhbited medicines in the cases of pregnancie. Ginekol. Polska.

[109] Ilyas R.A., Zuhri M.Y.M., Norrrahim M.N.F., Misenan M.S.M., Jenol M.A., Samsudin S.A., Nurazzi N.M., Asyraf M.R.M., Supian A.B.M., Bangar S.P. (2022). Natural fiber-reinforced polycaprolactone green and hybrid biocomposites for various advanced applications. Polymers.

[110] Tanha N.R., Nouri M. (2019). Core-shell nanofibers of silk fibroin/polycaprolactone-clindamycin: Study on nanofibers structure and controlled release behavior. Polym. Sci. Ser. A.

[111] Mohamadi P., Mirmoeini G., Bahrami H., Mohsenzadeh E., Cochrane C., Koncar V. (2022). Electrospinning of poly(caprolactone)/gelatin/clindamycin nanocomposites as an antibacterial wound dressing. Mat. Sci. Forum.

[112] Setia H., Javed M., Abdalkareem S., Abdelbasset K., Bokov D., Fakri Y., Najm M.A.A., Kazemnejadi M. (2022). Preparation of antibacterial gel/PCL nanofibers reinforced by dicalcium phosphate-modified graphene oxide with control release of clindamycin for possible application in bone tissue engineering. Inorg. Chem. Commun..

[113] Castillo-Ortega M.M., López-Peña I.Y., Rodríguez-Félix D.E., Del Castillo-Castro T., Encinas-Encinas J.C., Santacruz-Ortega H., Cauich-Rodríguez J.V., Quiroz-Castillo J.M., Chan-Chan L.H., Leyva-Verduzco I. (2022). Clindamycin-loaded nanofibers of polylactic acid, elastin and gelatin for use in tissue engineering. Polym. Bull..

[114] Vahedi M., Barzin J., Shokrolahi F., Shokrollahi P. (2018). Self-Healing, injectable gelatin hydrogels cross-linked by dynamic Schiff base linkages support cell adhesion and sustained release of antibacterial drugs. Macromol. Mater. Eng..

[115] Shekhawat D., Singh A., Bhardwaj A., Patnaik A. A short review on polymer, metal and ceramic based implant materials. Proceedings of the IOP Conference Series: Materials Science and Engineering.

[116] Janmohammadi M., Nazemi Z., Salehi A.O.M., Seyfoori A., John J.V., Nourbakhsh M.S., Akbari M. (2023). Cellulose-based composite scaffolds for bone tissue engineering and localized drug delivery. Bioactive Mat..

[117] Kołodziejska B., Kaflak A., Kolmas J. (2020). Biologically inspired collagen/apatite composite biomaterials for potential use in bone tissue regeneration—A review. Materials.

[118] Yue S., He H., Li B., Hou T. (2020). Hydrogel as a biomaterial for bone tissue engineering: A review. Nanomaterials.

[119] Ritschl L., Schilling P., Wittmer A., Bohner M., Bernstein A., Schmal H., Seidenstuecker M. (2023). Composite material consisting of microporous beta-TCP ceramic and alginate-dialdehyde-gelatin for controlled dual release of clindamycin and bone morphogenetic protein 2. J. Mater. Sci. Mater. Med..

[120] Kuehling T., Schilling P., Bernstein A., Mayr H.O., Serr A., Wittmer A., Bohner M., Seidenstuecker M. (2022). A human bone infection organ model for biomaterial research. Acta Biomater..

[121] Xin L. (2017). Preparation and characteristics of drug loaded PLGA chitosan/nano-hydroxyapatite membrane for guided periodontal tissue regeneration in surgical implanting. Acad. J. Second Mil. Med. Univ..

[122] Uskoković V., Desai T.A. (2014). In vitro analysis of nanoparticulate hydroxyapatite/chitosan composites as potential drug delivery platforms for the sustained release of antibiotics in the treatment of osteomyelitis. J. Pharm. Sci..

[123] Słota D., Florkiewicz W., Piętak K., Pluta K., Sadlik J., Miernik K., Sobczak-Kupiec A. (2022). Preparation of PVP and betaine biomaterials enriched with hydroxyapatite and its evaluation as a drug carrier for controlled release of clindamycin. Ceram. Int..

[124] Ben W., Sun P., Huang C.H. (2016). Effects of combined UV and chlorine treatment on chloroform formation from triclosan. Chemosphere.

[125] Bayston R., Ashraff W. (2012). Preventing infection on antimicrobial. Orthop. Proc..

[126] Antoniac I., Popescu D., Zapciu A., Antoniac A., Miculescu F., Moldovan H. (2019). Magnesium filled polylactic acid (PLA) material for filament based 3D printing. Materials.

[127] Pradid J., Keawwatana W., Boonyang U., Tangbunsuk S. (2017). Biological properties and enzymatic degradation studies of clindamycin-loaded PLA/HAp microspheres prepared from crocodile bones. Polym. Bull..

[128] Uskoković V., Hoover C., Vukomanović M., Uskoković D.P., Desai T.A. (2013). Osteogenic and antimicrobial nanoparticulate calcium phosphate and poly-(d,l-lactide-co-glycolide) powders for the treatment of osteomyelitis. Mat. Sci. Eng C.

[129] Vukomanović M., Škapin S.D., Jančar B., Maksin T., Ignjatović N., Uskoković V., Uskoković D. (2011). Poly(d,l-lactide-co-glycolide)/hydroxyapatite core-shell nanospheres. Part 1: A multifunctional system for controlled drug delivery. Colloids Surf. B.

[130] Vukomanović M., Škapin S.D., Poljanšek I., Žagar E., Kralj B., Ignjatović N., Uskoković D. (2011). Poly(d,l-lactide-co-glycolide)/hydroxyapatite core-shell nanosphere. Part 2: Simultaneous release of a drug and a prodrug (clindamycin and clindamycin phosphate). Colloids Surf. B.

[131] Vukomanović M., Zavašnik-Bergant T., Bračko I., Škapin S.D., Ignjatović N., Radmilović V., Uskoković D. (2011). Poly(d,l-lactide-co-glycolide)/hydroxyapatite core-shell nanospheres. Part 3: Properties of hydroxyapatite nano-rods and investigation of a distribution of the drug within the composite. Colloids Surf. B.

[132] Vukomanović M., Šarčev I., Petronijević B., Škapin S.D., Ignjatović N., Uskoković D. (2012). Poly(d,l-lactide-co-glycolide)/hydroxyapatite core-shell nanospheres. Part 4: A change of the surface properties during degradation process and the corresponding in vitro cellular response. Colloids Surf. B.

[133] Chen K., Guo B., Luo J. (2017). Quaternized carboxymethyl chitosan/organic montmorillonite nanocomposite as a novel cosmetic ingredient against skin aging. Carbohydr. Polym..

[134] Delir S., Sirousazar M., Kheiri F. (2020). Clindamycin releasing bionanocomposite hydrogels as potential wound dressings for the treatment of infected wounds. J. Biomat. Sci..

[135] Muhammad N., Siddiqua S. (2022). Calcium bentonite vs sodium bentonite: The potential of calcium bentonite for soil foundation. Mat. Today Proc..

[136] Idris S.A.S., Yucel O. (2022). Influence of bentonite nanoparticles on properties of PVP-CMC-gums hydrogel films for biomedical applications. Int. J. Eng. Sci. Technol..

[137] Bampidis V., Azimonti G., de Lourdes Bastos M., Christensen H., Dusemund B., Kos Durjava M., Kouba M., López-Alonso M., López Puente S., Marcon F. (2020). Safety and efficacy of sodium carboxymethyl cellulose for all animal species. EFSA J..

[138] Sadeghi S., Nourmohammadi J., Ghaee A., Soleimani N. (2019). Carboxymethyl cellulose-human hair keratin hydrogel with controlled clindamycin release as antibacterial wound dressing. Int. J. Biol. Macromol..

[139] Maver T., Mastnak T., Mihelic M., Maver U., Finšgar M. (2021). Clindamycin-based 3D-printed and electrospun coatings for treatment of implant-related infections. Materials.

